# Similarity between cutaneous reactions due to SARS-CoV-2 and its vaccinations

**DOI:** 10.2217/fvl-2021-0357

**Published:** 2022-09-23

**Authors:** Ömer Kutlu, Selami Aykut Temiz

**Affiliations:** ^1^Department of Dermatology & Venereology, Tokat Gaziosmanpasa University, School of Medicine, Tokat, Turkey; ^2^Department of Dermatology & Venereology, Necmettin Erbakan University, School of Medicine, Konya, Turkey

**Keywords:** COVID-19 vaccine, cutaneous reactions, SARS-CoV-2, skin

COVID-19, caused by SARS-CoV-2, continues to spread at full speed with its new variants including Omicron, while SARS-CoV-2 vaccination also continues at full speed. Currently, the most powerful tool in combating the COVID-19 pandemic is vaccination along with personal measures against SARS-CoV-2. During the 2 year pandemic period, it was observed that SARS-CoV-2 is associated with multiple system involvements, including the skin, apart from lung involvement. There are certain similar skin disorders that are both associated with the new coronavirus and its vaccines [1,2]. Fortunately, these reactions are commonly short, benign and self-limited. Herein, we have discussed the common cutaneous manifestations that are both associated with the new coronavirus and its vaccines.

## Chilblain-like lesions

Chilblain-like lesions (CLL) are mostly asymmetrical acral distributed in patients with COVID-19. It is a sign of mild-course COVID-19 and is predominant in younger patients. The localized acral skin damage may be the result of a strong immune-mediated reaction triggered by SARS-CoV-2. Therefore, patients with CLL have mild or no symptoms, negative RT-PCR and localized endothelial injury to the acral sites. The average occurrence time of lesions is about 10–14 days and lasts about 2 weeks, with spontaneous improvement [3,4]. There are numerous reports that revealed both virus related protein based weakened inactivated SARS-CoV-2 virus and mRNA vaccines including BNT162b2 (Pfizer-BioNTech) and mRNA-1273 (Moderna) can lead to CLL. The CLL may occur after the first and second doses of the vaccines. The onset of the lesions has been reported as 2–21 days [5,6]. Considering the characteristic formation and disappearance times of CLL lesions, no significant difference was found between the lesions that occurred after the COVID-19 and SARS-CoV-2 vaccination [7].

## Vasculitis

Vasculitis is another common cutaneous manifestation of the new coronavirus and its vaccines. As it is known, leukocytoclastic vasculitis can be associated with infections, drugs and vaccines. Similarly, there are numerous reports that revealed leukocytoclastic vasculitis is related to COVID-19 and its vaccines. The other reported vasculitic patterns that occurred after COVID-19 were small-vessel vasculitis, IgA vasculitis, granulomatosis with polyangiitis, eosinophilic granulomatosis with polyangiitis, purpura fulminans, urticarial vasculitis, lymphocytic and anti-neutrophil cytoplasmic antibody-associated vasculitis. The SARS-CoV-2 and its vaccines can imitate the self antigens of the body that resulted in immune dysregulation that resulted in vasculitis [1,2]. Most cases of COVID-19-related vasculitis run a mild to moderate course. The cornerstone of the medical treatment is systemic corticosteroids. Complete remission could be achieved in most patients [1].

## Morbiliform eruptions

Maculopapular rash, which also includes morbilliform eruptions, is the most common cutaneous side effect of the COVID-19 followed by CLL. The morbilliform eruption is also one of the common side effects of the COVID-19 vaccines and usually has a mild course [1,2]. Considering the characteristic formation and disappearance times of CLL lesions, there is also no significant difference between the lesions that occurred after the COVID-19 and SARS-CoV-2 vaccination.

## Pityriasis rosea & urticaria

We previously reported that there is an increased number of urticaria and pityriasis rosea (PR) during the COVID-19 pandemic when compared with the corresponding previous year of the current pandemic. Subsequent reports suggested that patients with COVID-19 who have PR-like dermatosis and urticarial rash include SARS-CoV-2 particles in their skin lesions [8]. Similarly, numerous cases contain reports that PR-like dermatitis and urticaria can be developed after COVID-19 vaccines. Of note, in our daily routine practice, we also encountered numerous cases of new urticarial eruptions after both inactivated and mRNA-based COVID-19 vaccines. As per our observations PR cases that developed after SARS-CoV-2 vaccinations were not demographically distinct from the ordinary and general PR. Interestingly, there is a higher incidence of PR cases among inactivated SARS-CoV-2 vaccines in the literature [1]. The exact effect of the differences should be illuminated with further studies.

## Herpes zoster

Herpes zoster can be triggered by COVID-19 vaccines. The occurrence of herpes zoster after the COVID-19 vaccine is mostly within 7–10 days. Interestingly, there is a report that even though a patient who has been vaccinated against the herpes zoster virus developed herpes zoster after COVID-19 vaccination. Similarly, to date, there are over 30 reports which suggested the possible relationship between COVID-19 and herpes zoster. The majority of cases of herpes zoster in patients with COVID-19 have a typical clinical presentation [9]. Considering the characteristic formation and disappearance times of herpes zoster lesions reveals similarity between cutaneous reactions due to SARS-CoV-2 and its vaccinations.

## Erythema multiforme

Many types of drug reactions including erythema multiforme, which developed after COVID-19, have been reported. Similarly, four cases of mRNA vaccine-related erythema multiforme have been recently documented [1,2]. Increased vaccine-mediated immune boosting may lead to delayed hypersensitivity reaction which is responsible for erythema multiforme like reactions.

## Other cutaneous manifestations

Apart from relatively common similar cutaneous manifestations of COVID-19 and its vaccine aforementioned above, there are certain rare skin disorders that can be seen as a result of both COVID-19 and its vaccine. These disorders can be listed as vesiculobullous eruptions, lichen planus, granuloma annulare, erythema annulare centrifugum, flare of herpes simplex, Rowell’s syndrome, DRESS syndrome, and recurrence of alopecia areata. Of note, to date, there are still no reports on the relationship between livedo reticularis-like eruption, one of the main serious cutaneous manifestations of the COVID-19 and COVID-19 vaccines.

In the early stages of the pandemic, cutaneous side effects of SARS-CoV-2 were described, while in the current stages of the pandemic, cutaneous side effects of the COVID-19 vaccine were described. In general, these cutaneous side effects are commonly temporary, benign, self-limited and generally not a contraindication to further doses of the vaccine. In this article, we would like researchers to pay attention to the similarity between cutaneous reactions due to SARS-CoV-2 and its vaccinations.

## Future perspective

The immune-based skin reactions due to both conditions may show that SARS-CoV-2 has a more immunological effect rather than infectious effect on the skin as in toxic shock syndrome. In addition, mRNA containing lipid nanoparticles found in COVID-19 vaccines can trigger autoimmunity by increasing the production of proinflammatory cytokines and chemokines. Furthermore, we know that the vaccines have mostly been developed against the antigenic Spike protein of the SARS-CoV-2 virus. Therefore, we would like to emphasize that the similarity of the skin reactions both after the COVID-19 and SARS-CoV-2 vaccinations may suggest the focus of Spike proteins of the virus in the pathogenesis of the skin-related diseases.

Skin manifestations associated with COVID-19 apart from livedo reticularis-like eruption are usually associated with milder disease, while it is not yet known whether the presence of post-vaccination skin findings is a strong or weak indicator of vaccine immunity. This condition is another gap that should be filled. As a result, further novel studies are required in order to find the exact pathogenesis of the cutaneous manifestations of SARS-CoV-2. In this context, addressing COVID-19 and its vaccine together may lead to new discoveries on the disease.

In the meantime, air pollution may increase the risk of getting coronavirus. Recent research from Rachel Nethery, Xiauo Wu, Francesca Dominici and other colleagues at Harvard showed that people who live in places with poor air quality are more likely to die from COVID-19 despite other factors such as pre-existing medical conditions, socioeconomic status and access to healthcare [10]. The comparison of cutaneous manifestations of vaccinations between the different air pollution areas may give certain indirect clues regarding the effects of COVID-19 and vaccines. In the next future, the effect of the different environmental conditions on the virus and vaccines may be important fields to deal with.
